# Low extracellular vesicle concentrations predict survival in patients with heart failure

**DOI:** 10.3389/fcvm.2023.1163525

**Published:** 2023-05-24

**Authors:** David Schöler, Sven H. Loosen, Theresa H. Wirtz, Jonathan F. Brozat, Lauredana A. dos Santos Ferreira Grani, Tom Luedde, Lisa Heinrichs, Derk Frank, Alexander Koch, Christoph Roderburg, Martina E. Spehlmann

**Affiliations:** ^1^ Clinic for Gastroenterology, Hepatology and Infectious Diseases, University Hospital Düsseldorf, Medical Faculty of Heinrich Heine University Düsseldorf, Düsseldorf, Germany; ^2^ Department of Gastroenterology, Digestive Diseases and Intensive Care Medicine, RWTH Aachen University Hospital, Aachen, Germany; ^3^ Department of Hepatology and Gastroenterology, Charite—Universitätsmedizin Berlin Campus Virchow-Klinikum (CVK) and Campus Charite Mitte (CCM), Berlin, Germany; ^4^ Internal Medicine III, University Hospital of Schleswig Holstein, Campus Kiel, Kiel, Germany

**Keywords:** extracellular vesicles, heart disease, survival, intensive care, biomarker, prognosis

## Abstract

**Background:**

Heart disease is of worldwide importance due to high morbidity and mortality. Extracellular vesicle (EV) concentration and size represent novel diagnostic and prognostic biomarkers, e.g. in patients with liver cancer, but data on their prognostic relevance in heart disease are lacking. Here, we investigated the role of EV concentration, size and zeta potential in patients with heart disease.

**Methods:**

Vesicle size distribution, concentration and zeta potential were measured by nanoparticle tracking analysis (NTA) in 28 intensive care unit (ICU) and 20 standard care (SC) patients and 20 healthy controls.

**Results:**

Patients with any disease had a lower zeta potential compared to the healthy controls. Vesicle size (X50) was significantly higher in ICU patients (245 nm) with heart disease as compared to those patients with heart disease receiving standard care (195 nm), or healthy controls (215 nm) (*p* = 0.001). Notably, EV concentration was lower in ICU patients with heart disease (4.68 × 10^10^ particles/ml) compared to SC patients with heart disease (7,62 × 10^10^ particles/ml) and healthy controls (1.50 × 10^11^ particles/ml) (*p* = 0.002). Extracellular vesicle concentration is prognostic for overall survival in patients with heart disease. Overall survival is significantly reduced when the vesicle concentration is below 5.55 × 10^10^ particles/ml. Median overall survival was only 140 days in patients with vesicle concentrations below 5.55 × 10^10^ particles/ml compared to 211 days in patients with vesicle concentrations above 5.55 × 10^10^ particles/ml (*p* = 0.032).

**Summary:**

Concentration of EVs is a novel prognostic marker in ICU and SC patients with heart disease.

## Introduction

Extracellular vesicles (EVs) are a heterogeneous group of small membrane-bound vesicles. EVs are further classified according to their size and biogenesis. While exosomes are rather small (100 nm in size) and are released upon exocytosis of multivesicular bodies (1), ectosomes or microparticles are somewhat larger (500 nm in size) and are derived from the cellular membrane (2). Finally, apoptotic bodies are >100 nm in size and are formed during the process of apoptotic cell death, as summarized in (2). EVs are released into the circulation by many different cell types, including immune cells (3), and contain a wide variety of biomolecules, including proteins, lipids, cytokines, hormones, as well as nucleic acids, mRNAs and non-coding RNAs (3). Recently, EVs have been proposed as mediators of cell-to-cell communication and the concentration / composition of EVs has been shown to change depending on the physiological state of the parent cells (2). The zeta potential is an electrostatic potential at the electrical bilayer surrounding a nanoparticle in solution (4). It has been shown to play a role in cell culture systems (5) and in the context of cardiac uptake and drug delivery (6). Circulating EVs and their cargo biomolecules have been identified as biomarkers and drivers of benign and malignant diseases including heart failure (HF).

HF is associated with structural and/or functional abnormalities of the heart. With a worldwide prevalence of 65 million affected patients, it represents a major global health burden (7). The clinical presentation and prognosis of patients with HF is largely dependent on ejection fraction. The mechanisms driving HF are heterogeneous and vary depending on the underlying disease etiology. Of note, EVs have been widely implicated in the pathophysiology of various cardiac diseases (8). For example, it has been suggested that EVs may act as intercellular drivers of cardiac inflammation, leading to cardiac injury and ultimately to HF (reviewed in 9,10).

In the present study, we aimed to evaluate the concentrations and sizes of circulating EVs as diagnostic and prognostic biomarkers in patients with HF at different stages of the disease. We provide evidence for a difference in EV concentrations in ICU and HF patients and a high prognostic relevance of EVs in HF.

## Patients and methods

### Study design

This exploratory observational study examines the role of EVs in patients with heart disease considering intensive care (ICU) or standard care (SC) therapy. 13 out of 28 patients treated in the ICU had heart disease and a further 20 patients with heart disease were treated with standard therapy. In addition, 20 healthy people were included as controls. Blood samples from the ICU patients were taken immediately after admission to the ICU. In the SC patients, date of blood analysis was variable, ranging from 98 days before to 14 days after admission. Blood samples were centrifuged at 2,000 G for 10 min and serum aliquots were stored at −80°C until analysis. The local ethics committees approved our study in accordance with the ethical standards of the Declaration of Helsinki (reference numbers EK 150/06 and AZ A 174/09).

In SC patients with heart disease, the diagnosis of dilated cardiomyopathy was confirmed by exclusion of stenosing coronary artery heart disease by coronary angiography, documented myocarditis, systemic disease (such as sarcoidosis), sustained arterial hypertension, or congenital malformation. In addition, 60% of patients had cardiac magnetic resonance imaging with gadolinium contrast and 50% underwent left ventricular biopsies to confirm the diagnosis. Patients had to have a left ventricular ejection fraction <30% as confirmed by echocardiography. Patients had to be at least 18 years old and able to give written informed consent. Control patients were healthy age- and sex-matched patients with no know cardiac or other major disease. Only 3 control patients were hospitalised. They were hospitalised because of unexplained dizziness and/or syncope, which was later classified as orthostatic dysregulation. All other controls were not hospitalised and were volunteered for the study. ICU patients were aged ≥18 years and had an ICU stay of ≥72 h. Written informed consent was obtained from all patients.

### Measurement of EV concentration and size

EV analysis was performed using the ZetaView multi-parameter Particle Tracking Analyzer (ParticleMetrix, Germany) to measure the size distribution and quantity of vesicles. The ZetaView technique is based on Brownian motion and this principle is used for the analysis of nanometre-sized particles (11,12). The accuracy of the ZetaView was assessed by performing auto-alignment prior to measurements using a standard calibration nanoparticle solution supplied by ParticleMetrix (diameter 110 nm). The camera focus was adjusted so that the particles appeared as sharp dots prior to the measurements. The sample expected to contain the highest number of vesicles was used to set the camera sensitivity, which was kept constant for subsequent measurements. Samples were diluted in particle-free PBS to achieve a particle count in the range of 1–9 × 10^7^ particles/mL (or approximately 200 particles per visual field, PVF). Using the script control function, three 30-second videos were recorded for each sample, with a sample advance and a 5-second delay between each recording.

### Statistical analysis

Statistical analyses were performed as recently described in detail (13). The Shapiro-Wilk test was used to test for normal distribution. Non-parametric data were compared using Mann-Whitney-U test and Kruskal-Wallis test. Paired samples were compared using the Wilcoxon signed-rank test. Box plots show medians, quartiles and ranges. Kaplan-Meier curves show the effect of EV concentration or size on overall survival (OS). The log-rank test was used to test for statistical differences. All statistical analyses were performed using SPSS 25 (SPSS, Chicago, IL, USA). A *p*-value of < 0.05 was considered statistically significant (* *p* < 0.05; ** *p* < 0.01; *** *p* < 0.001).

## Results

### Study cohort characteristics

The present analysis included 48 patients admitted to inpatient care (28 to ICU, 20 to SC) between 08/2009 and 07/2018. 20 healthy individuals were included as a control cohort. Overall, the cohorts (SC; ICU; without healthy controls) had a median age of 67 years (range 38–94.0) with a median BMI of 27 kg/m^2^ (range 16.5–58.6 kg/m^2^) and 65% (*n* = 31) were male. Of the 28 ICU patients included, 13 (46%) had documented heart disease, whereas all 20 SC patients had a history of heart disease. Heart disease was defined as HF with reduced ejection fraction (HFrEF). Notably, all patients in the SC group had a heart disease with dilated cardiomyopathy (DCM) and an ejection fraction <30%. The median age was well balanced between the cohorts. Of all 48 patients, *n* = 16 (33.3%) deceased during hospitalisation (*n* = 10) or follow-up (*n* = 6), 11 in the ICU and 5 in the SC group. The medium length of hospital stay for the entire cohort was 12 days (1–129). As expected, ICU patients received significantly longer medical treatment with a median hospital stay of 23 days (1–129) compared to SC patients (median 6 days, range 1–20) (*p* = 0.003). On average, patients spent 10.4 days in intensive care. A detailed description of baseline characteristics can be found in Table 1.

**Table 1:** Study cohort characteristics.

	All patients (ICU and SC)	ICU	SC	Controls
Number of patients	48	28	20	20
Sex				
Male, *n* (%)	31 (65)	17 (61)	14 (70)	14 (70)
Female, *n* (%)	17 (35)	11 (39)	6 (30)	6 (30)
Heart disease, *n* (%)	33 (69)	13 (46)	20 (100)	0 (0)
Age, median; mean	68.0; 66.9	69.5; 68.2	66.0; 65.2	64.0; 62.3
(range) [years]	(38.0–94.0)	(45.0–94.0)	(38.0–85.0)	(34.2–82.9)
BMI, median; mean	27; 29.2	26.0; 29.2	27.0; 29.2	25.5; 25.9
(range) [kg/m^2^]	(16.5–58.6)	(16.5–58.6)	(20.7–56.5)	(21.3–35.9)
Hospital days, median; mean	12; 19.3	23; 26.5	6; 8.7	4; 4.7
(range)	(1–129)	(1–129)	(1–20)	(4–6)
Deceased, *n* [%]				
Yes	16 [33]	11 [39]	5 [25]	0 [0]
No	32 [67]	17 [61]	15 [75]	20 [100]
Conc. of EV, median (range) [p/mL]	4.15 × 10^10^ (0.63–91)	3.3 × 10^10^ (0.86–91)	6.15 × 10^10^ (0.63–25)	1.06 × 10^11^ (0.19–4.6)
Size of EV, median (range) [nm]	218.4 (161.2–303.4)	235.7 (161.2–303.4)	187.0 (162.9–268.3)	202.2 (167.6–394.9)
Zeta Potential, median (range) [mV]	48.2 (−45.2–186.2)	39.0 (−45.2–186.2)	55.7 (−30.7–69.3)	62.9 (−38.1–167.1)

BMI, body-mass-index; EV, extracellular vesicles; p, particles.

### Patients with heart disease on SC have lower EV concentrations

**Figure 1:**
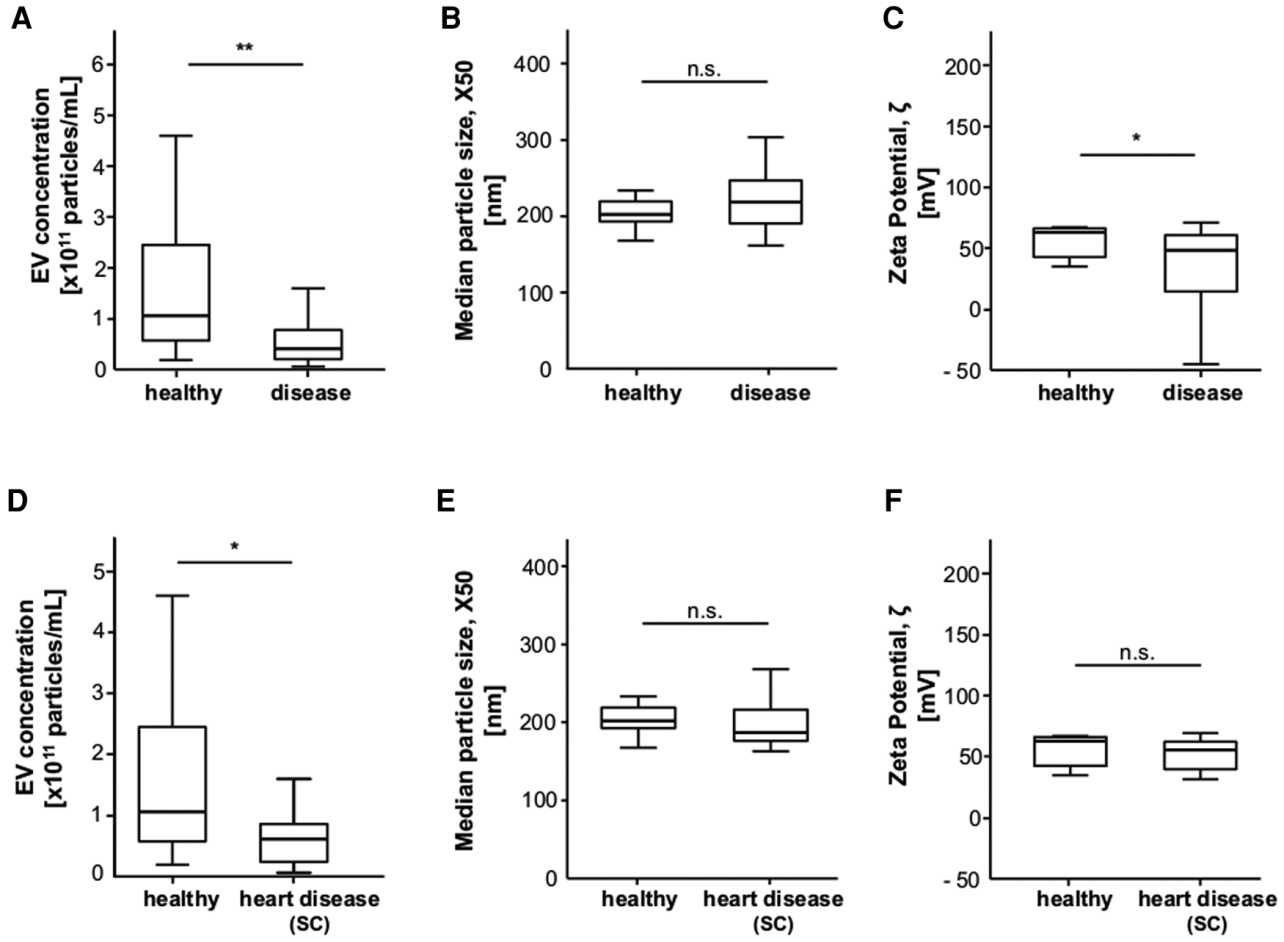
Patients with heart disease treated with standard care (SC) have significantly lower EV concentrations than healthy controls. (**A**) Patients with any disease have a significantly lower EV concentration than the healthy controls. (**B**) The median EV size is not different between patients with any disease and healthy controls. (**C**) Patients with any disease have a significantly lower zeta potential than the healthy controls. (**D**) Standard care patients with heart disease have a significantly lower EV concentration than healthy controls. (**E, F**) EV size and zeta potential are not different between standard care patients with heart disease and healthy controls.

To assess whether extracellular vesicles could play a role as potential biomarkers of disease, we measured EV concentration, size, and zeta potential in the different groups. The mean circulating EV concentration within the hospitalised patients (SC + ICU) was 8.1 × 10^10 ^p/ml (0.6–91), healthy controls had a mean EV concentration of 1.5 × 10^11^ (0.2–4.6) p/ml (*p* = 0.001) (Figure 1). Healthy controls had a mean vesicle size of 215.2 (167.6–394.9) nm and a zeta potential of 64.3 (−38.1–167.1) mV. The mean zeta potential in hospitalised patients with any disease was 42.4 mV, and the difference from healthy controls was significant (*p* = 0.023). Notably, patients with heart disease treated with standard therapy had significantly lower EV concentrations than healthy controls [on average 7.6 × 10^10 ^p/ml vs. 1.5 × 10^11^ (*p* = 0.03)].

Optimal cut-offs were found by using the web-application developed by (14). Four patients in the healthy control group were admitted as inpatients for evaluation, but no heart disease was found.

### ICU patients have lower EV concentrations, particle sizes and zeta potentials than healthy controls

**Figure 2:**
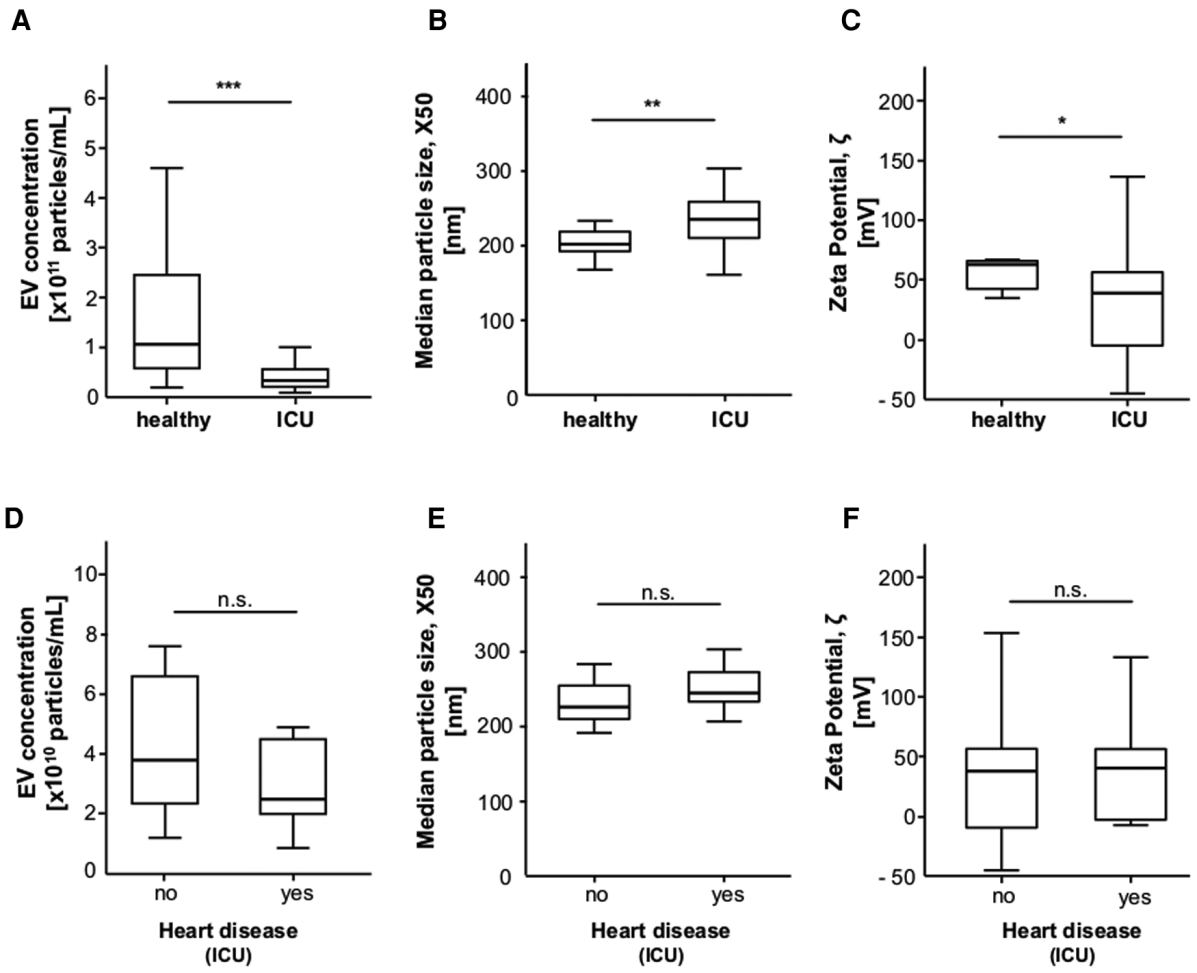
Ev concentration, size and zeta potential are significantly different between ICU patients and healthy controls. (**A**) ICU patients have a significantly lower EVs concentration compared to healthy controls. (**B**) ICU patients have a significantly higher EVs size compared to healthy controls. (**C**) ICU patients have a significantly lower zeta potential compared to healthy controls. (**D**) EVs concentration is lower in ICU patients with heart disease compared to ICU patients without heart disease, without reaching significance. (**E**) EVs size tends to be higher in ICU patients with heart disease compared to ICU patients without heart disease, without reaching significance. (**F**) Zeta potential is unchanged in ICU patients with heart disease compared to ICU patients without heart disease.

ICU patients had significantly lower EV concentrations than the healthy controls with a mean of 8.5 × 10^10 ^*p*/ml (0.9–91) vs. 1.5 × 10^11^ (0.2–4.6) p/ml (*p* < 0.001). Median particle size X50 was also significantly higher in ICU patients with 238.0 nm (161.2–303.4) vs. 215.2 nm (167.6–394.9) in the controls (*p* = 0.002). In addition, zeta potential was significantly lower in ICU patients at 40.0 mV (−45.2–186.2) (*p* = 0.021) (Figure 2). However, there was no significant difference in EV concentration, X50 and zeta potential between ICU patients with and without heart disease.

### EV concentration and EV size are significantly different between ICU patients with heart disease and healthy controls

**Figure 3:**
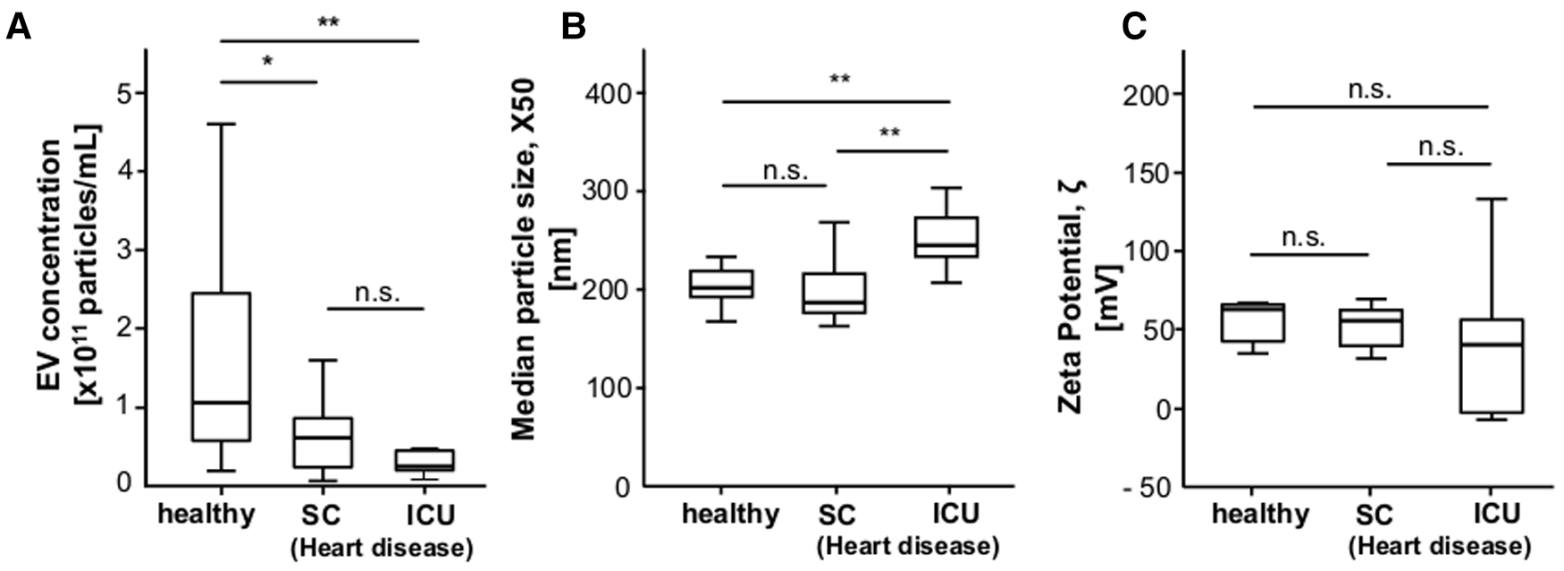
EV concentration and EV size are significantly different between ICU patients with heart disease and healthy controls. (**A**) Heart disease patients in ICU and SC have significantly lower EV concentrations than healthy controls. (**B**) Patients with heart disease in ICU have a significantly higher EV size than healthy controls and heart disease patients on standard care. (**C**) The zeta potential does not differ between patients with heart disease in ICU and regulare care and healthy controls.

Focusing on patients with heart disease, we found a significantly lower EV concentration in patients with heart disease on ICU (4.7 × 10^10 ^p/ml) and SC (7.6 × 10^10 ^p/ml) compared to controls (1.5 × 10^11^) (*p* = 0.002). In addition, ICU patients with heart disease had a significantly higher X50 (245 nm) than SC patients (195.2 nm) and controls (215.2 nm) (Figure 3).

## EV concentration is associated with survival probability in heart disease patients

**Figure 4:**
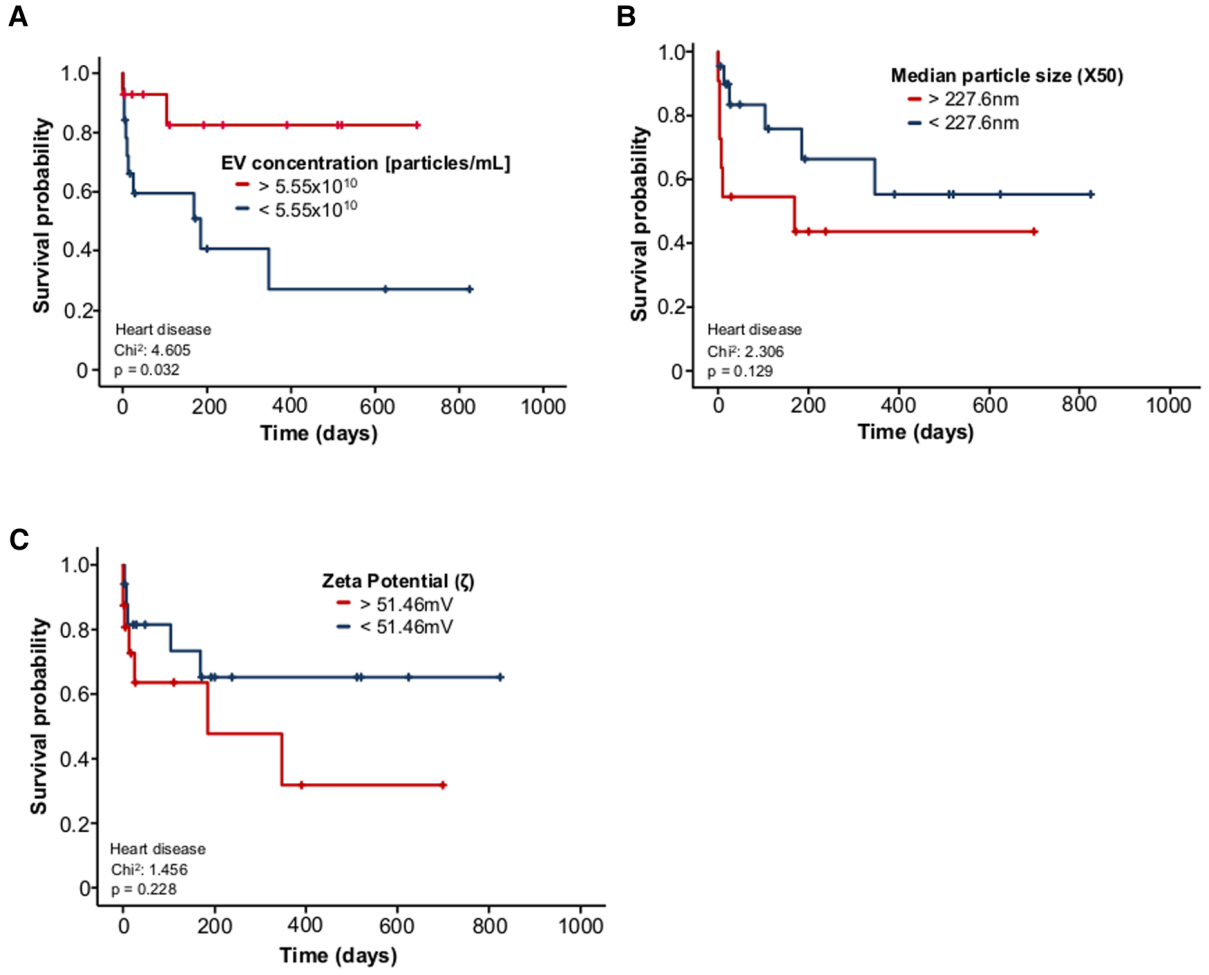
Reduced EV concentrations predict adverse outcome in patients with heart disease. (**A**) EV concentrations < 5.55 × 10^10^ particles/mL in patients with heart disease are associated with a significantly lower probability of survival than EV concentrations > 5.55 × 10^10^ particles/mL in patients with heart disease. (**B**) Survival is not significantly different between patients with an EV size > 227.6 nm and patients with EV size < 227.6 nm, although there is a trend towards worse survival in patients with EV size > 227.6 nm. (**C**) Patients with a zeta potential >51.46 mV do not have a significantly different survival probability than patients with a zeta potential <51.46 mV.

As patients with heart disease had lower baseline EV concentrations, we analysed the correlations with survival probability. Patients with EV concentrations <5.55 × 10^10^ particles/mL had a significantly lower survival than patients with EV concentrations >5.55 × 10^10^ particles/ml. Median overall survival was 140 days in patients with EV concentrations <5.55 × 10^10^ particles/ml vs. 211 days in patients with EV concentrations >5.55 × 10^10^ particles/ml (*p* = 0.032) (Figure 4).

## Discussion

In Germany, the treatment of intensive care patients accounts for a large proportion of health care costs (15). Costs are rising due to the increasing number of elderly patients with multiple comorbidities who require complex and technologically advanced therapy. The cost per ICU patient depends on the severity of the illness and the length of stay in the ICU (16). HF is the third leading cause of death wordwide and accounts for a significant proportion of ICU admissions: 14% of patients are admitted to a cardiac ICU with a diagnosis of HF and a further 5% for cardiogenic shock. Due to the longer length of stay, HF patients accounted for 33% of total ICU patient-days. Furthermore, HF patients are at high risk of death and require the most complex and resource-intensive care in the ICU (17). Therefore, it is necessary to gain a better understanding of the pathophysiological processes in ICU patients and HF and to develop prognostic markers for these diseases.

In this study, we analysed extracellular vesicle concentrations and sizes and their prognostic and predictive potential in ICU patients, patients with severe HF (LVEF <30%) due to dilated cardiomyopathy, and an age- and sex-matched healthy control cohort. We show that vesicle concentrations were significantly lower in ICU patients compared to healthy controls. In contrast, median particle size X50 was significantly higher in ICU patients, while zeta potential was lower in ICU patients. Studies have shown that EV can mediate either pro- or anti-inflammatory signals, depending on the underlying disease and disease stage (18). Furthermore, circulating EVs play an important role in acute lung injury. Serum EVs from a model of sepsis-induced acute lung injury were taken up into the lung parenchyma of naive mice and increased the number of total and activated alveolar macrophages. This cellular effect may be due to the effects of miRNA miR155, which is involved in the regulation of inflammatory responses mediated by immune cell infiltration (19,20). The lack of high numbers of EVs in ICU patients in our study may indicate a deficiency to mediate sufficient immune system activation in response to sepsis and lung injury. On the other hand, EVs may also be beneficial in lung injury. Treatment with human MSC-derived EVs ameliorates decreased alveolar septation, pulmonary hypertension, and fibrosis in a hypoxia-induced lung injury model (21).

We found that EV size and zeta potential were significantly different in ICU patients. Zeta potential is a popular method to measure the surface potential of EVs, while being used as an indicator of surface charge and colloidal stability. To date, there is limited data on zeta potential in ICU and HF patients. Several groups have investigated the size of EVs in sepsis and other inflammatory diseases. A study in mice showed that EV size is reduced in septic mice (22). Notably, our research group has found that enlarged extracellular vesicles are a negative prognostic factor in patients undergoing TACE for primary or secondary liver cancer (23). As EV size depends on the cell of origin of the vesicles, further investigations are needed to determine the impact of different EV sizes in our study of ICU patients.

We also found that heart disease patients on ICU and SC had significantly lower EV concentrations than healthy controls, and we also showed that heart disease on SC had significantly lower EV concentrations than healthy controls. Notably, patients with heart disease in the ICU had a significantly higher EV size than healthy controls and patients with heart disease in the SC, but zeta potential did not differ between patients with heart disease in the ICU and SC and healthy controls. While extracellular vesicles mediate physiological cardiomyocyte functions, they also alter cellular functions under pathological conditions and promote cardiovascular disease (24). EVs released from rat cardiomyocytes *in vitro* showed a strong increase in concentration in response to hypoxia (25). EVs secreted by hypertrophied cardiomyocytes contain heat shock protein 90 (HSP90) and interleukin (IL-6) signal transducer and activator of transcription 3 (STAT3) pathway signalling (26). Given the potent anti-inflammatory effects of STAT3, it is possible that patients with HF may have a lower level of STAT3 activation due to their reduced levels of EVs. A study in mice subjected to HF by transverse aortic constriction showed that the animals had increased levels of EVs, which transfer the angiotensin II type 1 receptor to the myocardium released into the serum (27). Analysis of cardiac exosomes from failing hearts compared to exosomes from normal hearts showed that exosomes from failing hearts exacerbated cardiac function and left ventricular remodelling, presumably due to dysregulation of miR-21–5p in these EVs (28). In contrast, EVs also show therapeutic immunomodulatory properties, improving survival and myocardial depression, as well as prolonging survival in a model of sepsis (29).

We also found that EV concentrations <5.55 × 10^10 ^particles/ml were associated with a significantly lower survival probability than EV concentrations >5.55 × 10^10 ^particles/ml in patients with heart disease. However, neither EV size nor zeta potential had a significant effect on survival. EVs have the ability to transfer their signalling cargo into endothelial cells, vascular smooth muscle cells, or into atherosclerotic plaques, which contribute to the progression of cardiovascular disease. It is therefore conceivable that circulating levels of EVs may be associated with the occurrence of major adverse cardiac events (30). Most of the studies show a positive correlation between EV counts and acute cardiovascular disease, regardless of the cell type of origin. This observation may be due to an increase in EV production after cell activation (31). However, large randomised controlled trials are still lacking in this area. In a small study, EV levels were measured in different cell types in patients at risk of cardiovascular disease who consumed a Mediterranean Diet. Participants who suffered a cardiovascular event such as myocardial infarction, stroke or death from cardiovascular disease within one year showed increased EV release from lymphocytes and smooth muscle cells. Participants who did not have a future cardiovascular event showed decreased EV release from these cells (32). Quantitative measurements of CD31/Annexin A5-positive EVs were also taken in patients with stable coronary artery disease. EV levels were higher in patients who went on to develop a major adverse cardiovascular or cerebral event (30). This study claims that diabetes and male sex are positively correlated with the number of EVs. However, we did not find a significant correlation with EV levels in our patients. A single-centre cohort of 1,060 patients found that the protein content of EVs was associated with an increased risk of secondary cardiovascular events. Specifically, increased levels of cystatin C, serpin F2, and CD14 were correlated with an increased risk of myocardial infarction, vascular events, and mortality. Therefore, the proteins cystatin C, serpin G1 and F2, and CD14 were identified as potential biomarkers of survival in this study (33). We acknowledge that our studies need to be further specified by characterisation of EV contents such as nucleic acids and proteins.

In summary, the data included in this study support the conclusion that concentration, size and zeta potential are significantly different between ICU patients and healthy controls. In patients with HF, EV concentration, but not size and zeta potential, are significantly different from healthy controls. Importantly, EV concentration has a strong prognostic potential for survival in patients with HF. We recognize the limitations of our analyses. Most importantly, the number of patients included in our study is small and patients were enrolled in only two centres. In addition, the timing of blood analysis in the SC cohort was variable, which introduces a potential bias. ICU patients with heart disease have a trend towards a lower vesicle concentration as compared to ICU patients without heart disease (Figure 2D), although not reaching significance. The number of ICU patients was relatively small, which must be taken into account when interpreting the association of EV concentration/size and zeta potential with HF in this group. Finally, our experiments did not purify EVs or exosomes or include EV contents such as nucleic acids and proteins. Therefore, larger and more elaborate studies are needed to characterise EVs in ICU and HF patients after purifying and characterizing exosomes and exclusion of contamination, e.g., by apolipoproteins. The limited availability of patient sera in this study precluded these important analyses. Nevertheless, our study is the first to provide evidence for a significance of EV concentrations in ICU and HF patients and a high prognostic relevance of EVs in HF.

## Data Availability

The data analyzed in this study is subject to the following licenses/restrictions: Data included into this analysis represent highly sensitive medical data. It is directly against German (and European) law to publish such data in a way that would allow identifying individual patients (e.g. by providing different clinical values of one distinct patient). However, the data that support the findings of this study are available on request from the corresponding author and the Clinic for Gastroenterology, Hepatology and Infectious Diseases, University Hospital Düsseldorf, Medical Faculty of Heinrich Heine University Düsseldorf, Moorenstraße 5, 40225 Düsseldorf, Germany (Wissenschaft.Gastro@med.uni-duesseldorf.de). Requests to access these datasets should be directed to David Schöler, Wissenschaft.Gastro@med.uni-duesseldorf.de.
